# Role of Fibrinogen in Type-2 Diabetes Mellitus with Diabetic Neuropathy and its Preliminary Mechanism

**DOI:** 10.2174/0929866530666230509140515

**Published:** 2023-07-14

**Authors:** Wei-Li Gu, Zhen-Hong Li, Si-Qin Zhang, Pian Ao, Xiao-Bei Zhu, Xin Zhao, Xin-Yue Zhang, Deng-Feng Zhang, Xiao-Juan Huang, Yu Jiang, Li Wei

**Affiliations:** 1 College of Basic Medicine, Guangxi Medical University, Nanning, 530021, China;; 2 Department of Ultrasound Diagnosis, 923 Hospital of the People’s Liberation Army, Nanning, 530021, China

**Keywords:** Fibrinogen, diabetic neuropathy, demyelination, inflammation, blood coagulation, vascular stenosis

## Abstract

**Introduction:**

Diabetic peripheral neuropathy (DN) is the most common complication of type 2 diabetes mellitus (T2DM).

**Objective:**

This study aimed to explore the role of fibrinogen (FIB) in T2DM neuropathy and its preliminary mechanism.

**Methods:**

Ten male Sprague-Dawley rats were divided into a normal control group (NC group) and a T2DM neuropathy model group (DN group). The DN group was given a high-energy diet and streptozotocin, while the NC group was given a normal diet and a citric acid buffer. The expression levels of related proteins were analysed.

**Results:**

Electrophysiology: Compared with the NC group, the conduction latency of the somatosensory-evoked potential and nerve conduction velocity was prolonged in the DN group, while the motor nerve action potential was decreased. As seen under a light microscope, the peripheral nerve fibres in the DN group were swollen, and the nerve fibres in the posterior funiculus of the spinal cord were loose or missing. Moreover, as seen under an electron microscope, the peripheral nerve demyelination of the DN group was severe, with microvascular blood coagulation, luminal stenosis, and collapse. Compared with the NC group, in the DN group, the expression of FIB was positively correlated with the expression of both ionised calcium-binding adaptor molecule-1 and glial fibrillary acidic protein. Compared with the NC group, in the DN group, the expression of platelet/endothelial cell adhesion molecule-1 and B-cell lymphoma 2 was negatively correlated.

**Conclusion:**

The increased concentration of FIB may be the cause of neuropathy, and its mechanism may be related to its promotion of inflammatory response, blood coagulation, and vascular stenosis.

## 1 INTRODUCTION

Diabetes is extremely harmful to the human body, and diabetic peripheral neuropathy (DN) is the most common complication of type 2 diabetes mellitus (T2DM), leading to high morbidity and mortality rates [1, 2]. The lesions can involve the sensory nerves, motor nerves, and autonomic nerves, with a disease rate of more than 60%. At present, neuro-electrophysiological testing is commonly used to assess the neurological functioning of patients with diabetes. Nerve conduction velocity (NCV) examination is a common method for quantitatively checking peripheral nerve function in such patients and is considered to be the gold standard for the diagnosis of DN [3].

Zhou *et al.* [4] have found that diabetes can increase the level of fibrinogen (FIB) (*i.e.* coagulation factor I) in the body, and although FIB is closely related to degenerative changes in nerves, its specific mechanism is not clear. Fibrinogen is a macromolecular glycoprotein synthesised by the liver, and it has the highest blood content of all coagulation factors. It plays a dynamic balancing role in blood coagulation and fibrinolysis, maintains normal blood circulation, and plays a role in blood vessels functioning by regulating coagulation, platelet aggregation and vascular endothelial cell function [5]. A high concentration of FIB in plasma causes the blood to become hypertonic, increasing its viscosity and reducing its fluidity, easily leading to thrombosis. Furthermore, the microvessels become ischaemic and hypoxic due to the occurrence of thrombosis. The microcirculation is destroyed, and the nutritional state of the peripheral nerve tissue deteriorates [4]. In addition, an increase in FIB levels leads to the destruction of vascular endothelial cell function and an increase in vascular permeability as well as other pathological changes that strengthen the inflammatory response and aggravate the condition [6]. Moreover, it has been reported that leukocyte- and platelet-rich fibrin, a second-generation platelet concentrate, is heavily used in blood coagulation and tissue repair due to its angiogenic and wound-healing properties, which also provides crucial evidence of FIB in the process of blood coagulation and tissue repair [7].

Studies have found that cognitive dysfunction and even dementia can be serious complications of T2DM [8]. The hippocampus mediates cognitive functions, such as learning and memory, and insulin and insulin receptors are selectively distributed throughout the hippocampus, cerebellum, and other tissues of the central nervous system [9]. Hippocampal insulin resistance leads to mitochondrial dysfunction and increased reactive oxygen production, and impaired hippocampal insulin signal transduction may lead to cognitive dysfunction [10, 11]. Studies have shown that learning and memory impairment in T2DM rats is related to the weakening of the long-term potentiation effect of dentate gyrus granule cells and pyramidal cells in the CA1 area of the hippocampus [12]. Many studies have reported on DN. However, neuropathy in patients with T2DM is not limited to the peripheral nerves. It can be seen that central neuropathy is also a serious complication for patients with T2DM.

In the present study, Sprague-Dawley (SD) rats with similar clinical structures and functions are selected to create animal models of T2DM neuropathy. The aim is to simulate the characteristics of clinical neuropathy to investigate its pathogenesis, diagnosis, and early intervention. The modelling methods for T2DM neuropathy include gene knockout, diet induction, transgenic technology, and experimental induction [13]. The present study uses an experimental induction method, *i.e.* a high-energy diet combined with streptozotocin (STZ) induction. The advantages of this method are high modelling rates, low mortality rates, strong reproducibility, consistent onset time, and moderate to high blood sugar. [14]. Streptozotocin acts on and destroys islet β cells and the function of pancreatic islets in animal models according to the injection dose [15]. High-dose injections of STZ can increase blood sugar in animal models, but there is no significant neuropathy [16]. Low-dose STZ injections (< 50 mg/kg) gradually increase the blood sugar levels in animal models and gradually destroy the islet β cells, which is consistent with the characteristics of T2DM.

In this work, we investigate the neurophysiological and pathomorphological results in T2DM rat models and explore the correlation between DN and the expression of FIB. In addition, we evaluate the correlation between FIB and several specific markers, such as glial fibrillary acidic protein (GFAP), which signifies astrocyte activation, ionised calcium-binding adaptor molecule-1 (IBA1), a microglia-/macrophage-specific protein with increased expression during the activation of these cells, and platelet/endothelial cell adhesion molecule-1 (CD31). Briefly, the aim of this study is to investigate the role of FIB in T2DM with diabetic neuropathy and its preliminary mechanism *via* neuro-physiological, pathomorphological, and immunohistochemi-cal approaches to provide a new reference for the treatment of diabetic neuropathy.

## 2 MATERIALS AND METHODS

### 2.1 Experimental Methods

#### 2.1.1 Animals and Groups

The Experimental Animal Center of Guangxi Medical University purchased 20 SPF-grade SD male rats weighing 229.6 ± 8.50 g (animal production licence number: SYXK [Guangxi] 2020-0004). The temperature of the animal room was 20°C~26°C, and the daily temperature difference was <4°C. The relative humidity of the animal room was 40%~70%, and the environment was one of natural light that alternated between daytime and nighttime. Strict inspection and sanitation procedures were performed once a day. The experiment was approved by the Animal Ethics Committee of Guangxi Medical University (experiment approval no. 202006013) and was strictly bound by the national ethics and use guidelines for experimental animals.

The purpose of purchasing 20 rats was to avoid injury or death of the rats caused by unexpected factors in the feeding process. In fact, after the establishment of the T2DM rat model, five rats from the normal control group (NC group) and five rats from the T2DM neuropathy model group (DN group) were randomly selected for the experiment. Therefore, 10 rats from the two groups were used for the electrophysiological and immunohistochemical tests.

The animals were fed three times a day, with unlimited water intake. In order to avoid injury or death of the rats caused by unexpected factors in the feeding process, we established the TPDM rat model with 15 rats. After one week of adaptive feeding, the rats were divided randomly into two groups: the NC group (n = 5) and the DN group (n = 15). The rats in the NC group were fed an ordinary diet, while the rats in the DN group were fed a high-sugar and high-fat diet (Boaigang-B1135DM, Boaigang Biotechnology Co. Ltd, Beijing, China) for four weeks. The high-sugar and high-fat feed comprised the classic formula of 66.5% ordinary feed, 10% lard, 20% sucrose, 2.5% cholesterol, and 1% sodium cholate.

Four weeks later, after having fasted for more than 12 hours, the DN group was injected intraperitoneally with 25 mg/kg STZ (Sigma, St. Louis, MO, USA) once a day for two days [16]. The NC group was injected intraperitoneally with the same amount of citric acid buffer. Seven days later, according to random blood glucose levels of > 13.8 mmol/L, 5 rat models from the original 15 rats were included in the DN group (n = 5). The results of the electrophysiology and histopathology tests confirmed the success of the T2DM neuropathy model.

#### 2.1.2 Electrophysiological Testing

All rats were weighed and anaesthetised on an empty stomach. The standard specification was 10% chloral hydrate at 40 mg/kg. The internal temperature of the laboratory was maintained above and below 28°C. A VikingQuest electromyography instrument from the Thermo Nicolet Corporation (USA) was used [17].

1. Motor conduction velocity (MCV) detection using the rats’ femoral nerves: The stimulation electrode, recording electrode, and reference electrode all used a subcutaneous needle electrode (Thermo Nicolet Corporation). The detection parameters were compound muscle action potential (CMAP) and distal movement latency (DML). The method of operation was as follows: the rats were placed in a supine position, and the recording electrode was placed on the rectus muscle, *i.e.* the midpoint of the connection between the inguinal ligament and the patella. The reference electrode was placed above the patella. The stimulating electrode was placed in the femoral triangle area (located outside the femoral artery), and the cathode was placed close to the recording electrode. The DML and CMAP amplitudes were recorded.
2. Somatosensory-evoked potential (SEP) detection of the lower limbs: The stimulation electrode, recording electrode, and reference electrode all used a subcutaneous needle electrode (Thermo Nicolet Corporation). The rats were placed in the prone position, and the stimulation point was at the medial malleolus of the right tibial nerve. The stimulation intensity was appropriate when there was a slight twitch of the toes. The recording point was the apex of the contralateral cerebral cortex, level with the lower line of the ear. Record waveform: apex (cortex): P40 potential.

#### 2.1.3 Rat Tissue Specimen Collection

Various tissues were removed from both groups of rats. To comprehensively evaluate the neural function of the T2DM rat models, the removed parts included the central nerve and peripheral nerve, including the cerebral hippocampus, spinal cord, and femoral nerve. To start, all rats were weighed and anaesthetised by intraperitoneal injection under fasting conditions. The standard specification was 10% chloral hydrate at 40 mg/kg. The rats were euthanised *via* the acute massive blood loss method and placed in an icebox. The skin and fascia were quickly cut, and the tissue foreign bodies were washed with 0.01 mmol/L pre-cooled sterile phosphate-buffered saline (PBS; cat. no. Solarbio P1010, Beijing Suolaibao Technology Co., Ltd). The femoral nerve was immersed in a 3% glutaraldehyde solution to prepare for viewing under an electron microscope. The rest of the tissues were frozen at −80°C.

#### 2.1.4 Luxol Fast Blue Staining

Luxol fast blue staining was performed using a Luxol Fast Blue Kit (cat. no. Solarbio G1120, Beijing Suolaibao Technology Co., Ltd). The procedure was performed as previously described [18]. The slices were incubated in Luxol fast blue staining solution at room temperature for 24 hours and differentiated in 0.05% lithium carbonate solution; then, 70% ethanol was added until the grey matter and white matter were clear. The sections were counterstained with 0.1% cresyl violet staining solution, dehydrated, and fixed with permanent fixation medium.

#### 2.1.5 Nissl Staining

Nissl staining was performed to assess neuronal survival. The procedure was performed as previously described [19]. The slices were washed with deionised water and dipped in a warm solution of 1% thionine for 45 min before being differentiated with 70% alcohol for about 5 min.

#### 2.1.6 Haematoxylin and Eosin Staining

A haematoxylin and eosin (H&E) staining kit was used for the staining (cat. no. Solarbio G1120, Beijing Suolaibao Technology Co., Ltd). The procedure was performed as previously described [20]. Tissues were dehydrated using an ethanol gradient, paraffin-embedded, and sectioned at 5 μm thickness. The sections were then deparaffinised with xylene, hydrated with an ethanol gradient (100%, 100%, 95%, 85%, 75%, and 50%), stained with haematoxylin for two minutes, differentiated with 1% hydrochloric acid ethanol for three seconds, stained with eosin for two minutes and rinsed in running water. Finally, the tissues were dehydrated in xylene and an ethanol gradient before being fixed with Cytoseal 60.

#### 2.1.7 Analysis of Proteins FIB, GFAP, IBA1, Bcl-2 and CD31 Expression by Immunohistochemical Staining

Immunohistochemical staining was performed as previously described [21]. Tissue sections (4 μm thick) were dewaxed in dimethylbenzene. Next, the slices were immersed in 3% hydrogen peroxide for 15 min and washed three times in 0.01 mol/L PBS at pH 7.4 for 5 min each time. After 20 min of blocking in serum buffer at room temperature, the sections were incubated overnight with primary antibody (rabbit monoclonal to IBA1, cat. no. ab178846, 1:2,000; Bcl-2, cat. no. ab182858, 1:500; and CD31, cat. no. ab28364, 1:50; rabbit polyclonal to GFAP, cat. no. ab7260, 1:2,000; and FIB, cat. no. ab2413, 1:500; Abcam Company, UK) at 4°C. Next, the sections were rinsed three times in PBS for five minutes each time and incubated with fluorescent-labelled secondary antibody at room temperature for one hour. Then, the sections were stained with 3,3’-diaminobenzidine or counterstained with DAPI. The stained sections were observed and images were captured using an optical microscope or a laser-scanning confocal microscope (Olympus, Tokyo, Japan). Finally, the images were evaluated using the Image-Pro Plus 6.0 software (Media Cybernetics, CA, USA) according to the protocol described in a previous study [22].

### 2.2 Statistical Methods

For the image analysis of experimental tissues, an ImageJ high-definition colour pathological graphic analysis system was used to analyse the sections of each group for tissue staining, and the specific values of each group were detected. This study used the SPSS 22.0 statistical software for analysis, and the measurement data distribution was expressed as the mean ± standard deviation (±s). The data results met the standard of normal distribution. Specific comparisons between the two groups were carried out using the Student-Newman-Keuls method. The standard of significance was *α* = 0.05. A value of *P* < 0.05 indicated that the difference was statistically significant, while a value of *P* < 0.01 indicated that the difference was extremely statistically significant.

## 3 RESULTS

The results of the electrophysiological testing and pathological staining of the rats confirmed the successful establishment of the T2DM neuropathy rat model. Furthermore, the immunostaining results revealed that in the T2DM neuropathy rats, the expression of FIB, GFAP, and IBA1 was upregulated, and the expression of CD31 and Bcl-2 was downregulated. It was found that FIB was positively correlated with the expression of inflammatory factors GFAP and IBA1 and negatively correlated with the expression of CD31 and Bcl-2. Overall, the results suggested that neurodegenerative changes in T2DM may be caused by the interaction between FIB and inflammatory factors.

### 3.1 The Successful Establishment of the T2DM Neuropathy Rat Model

**Figure 1:**
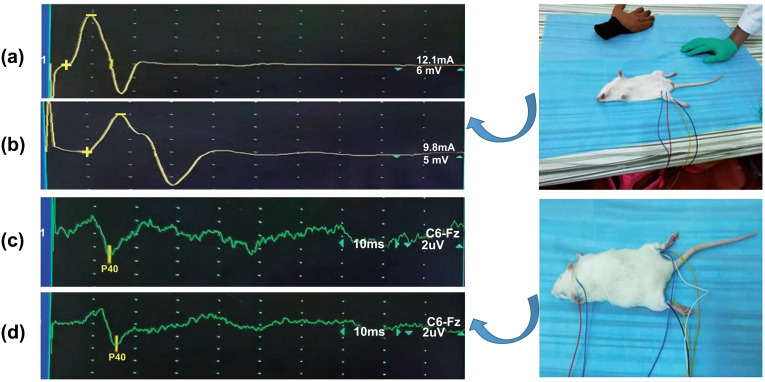
Electrophysiological test in rats. (**a, b**) The muscle action potential is generated that is used to measure the MCV of the femoral nerve. (**c, d**) Lower limbs somatosensory-evoked potential trace. **a, c**: NC group; **b, d**: DN group.

**Figure 2:**
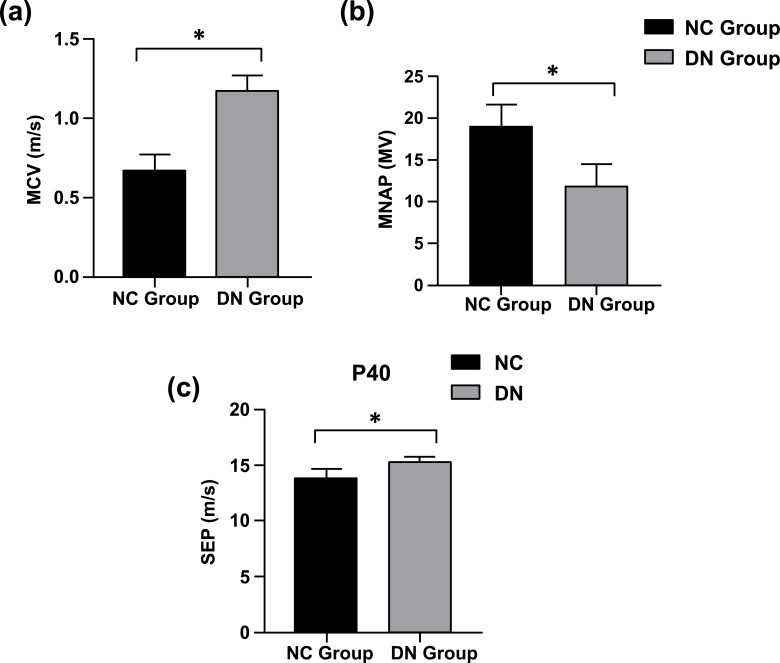
MCV, MNAP, SEP Comparison of Rat in Two Groups. (**a**) Quantitative comparison of MCV between two groups in m/s. (**b**) Comparison of MNAP average peak value in *mV*. (**c**) Comparison of the P40 peak of lower limbs SEP in ms. **Note:** compared with NC *P < 0.05.

**Figure 3:**
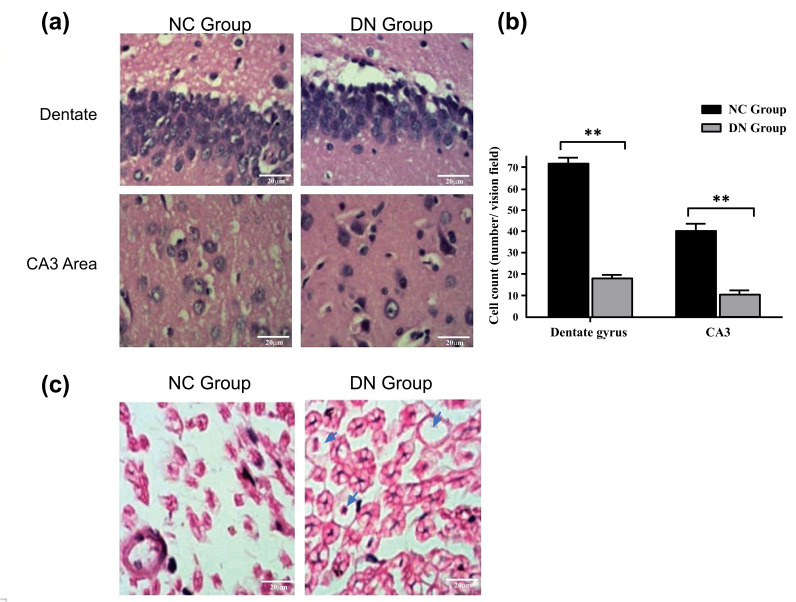
The hippocampal neurons (HE staining, magnification, ×40). (**a**) The number of neurons in the dentate gyrus, CA3 of the hippocampus in NC and DN group. (**b**) The number of neurons in the hippocampus. (**c**) HE staining of femoral nerve cells in NC and DN group, the blue arrow indicates that the femoral nerve in the DN group is vacuolated after demyelination. **Note:** **:*P* < 0.01.

According to Figures (**1** and **2**), the latency of MCV in the DN group was prolonged in comparison with that in the NC group (NC, n = 4, 0.68 ± 0.10; DN, n = 4, 1.18 ± 0.10; *P* < 0.05) (Figures **1a,b** and **2a**). Similarly, as in Figures (**1** and **3**), we observed the same trend in that the latency of SEP in the DN group was prolonged compared with that in the NC group (NC, n = 4, 13.83 ± 0.85; DN, n = 4, 15.28 ± 0.50; *P* < 0.05) (Figures **1c,d** and **3c**). However, compared with the NC group, the motor nerve action potential in the DN group decreased, as shown in (Figure **2b**) (NC, n = 4, 18.97 ± 2.69; DN, n = 4, 11.82 ± 2.66; *P* < 0.05). These results indicated that peripheral nerve demyelination and axon injury occurred in the DN group. According to the results of the H&E staining, as seen under a light microscope, compared with the NC group, the H&E staining of the central hippocampal nerve in the DN group revealed that the number of neuronal cells decreased and that the nucleus underwent pyknosis (Figures **3a** and **b**). The H&E staining of the femoral nerve showed that the axons of the myelinated nerve fibres in the NC group were dark, located in the centre of the myelin sheath, and surrounded by myelin; the myelin sheath had a network shape. In the DN group, the myelinated nerve fibres were swollen, and the myelin sheath, which had a foamy shape, was gone (Figure **3c**). The above results suggested that the neuronal cells in the DN group underwent harmful changes, including deletion, degeneration, and apoptosis.

**Figure 4:**
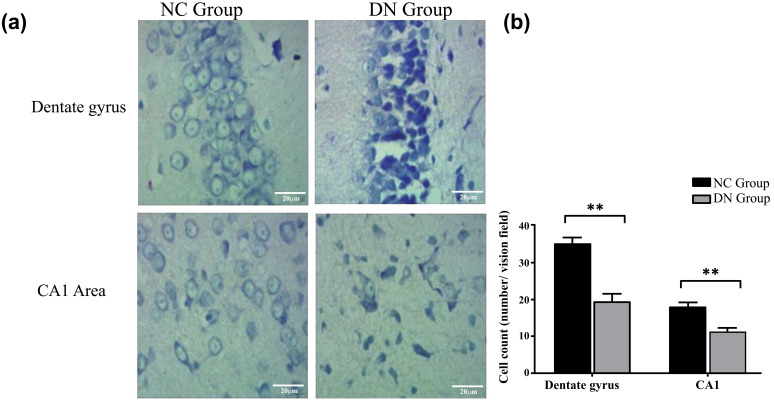
Number of cells in the hippocampus (per field) (n = 5). (**a**) Nissl staining in hippocampus dentate gyrus and CA1 area. (**b**) The Quantification of Nissl staining in hippocampus dentate gyrus and CA1 area. **Note:** **:*P* < 0.01.

Similarly, from the observation of the Nissl staining under a light microscope, the hippocampal neurons in the NC group were round and polygonal, the nucleus was vacuolar and centred, and the cytoplasm contained blue, patchy Nissl bodies (Figure **4a**). In the DN group, the Nissl bodies were dissolved, the neuron cell body was swollen, and the nucleus deviated and underwent pyknosis. The nucleolus was obvious, and the Nissl bodies were transparent after dissolution (Figure **4a**). The number of hippocampal neurons in the DN group was significantly lower than that in the NC group (*P* < 0.01) (Figure **4b**). The Nissl staining results showed that the neuronal cells in the DN group had undergone harmful changes, such as degeneration and apoptosis.

**Figure 5:**
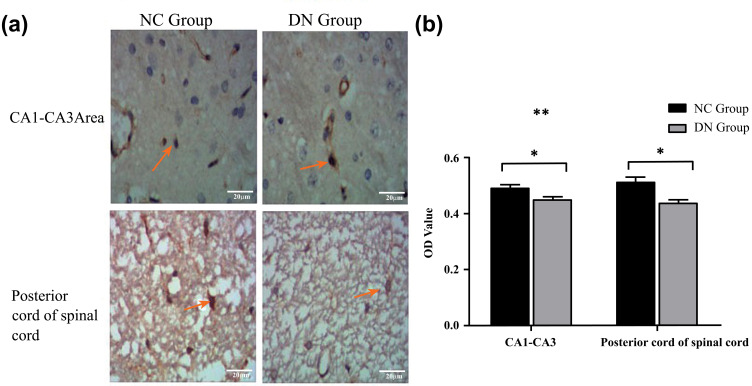
The expression of Bcl-2 in the hippocampus and spinal cord of the two groups of rats. (**a**) Expression of hippocampus CA1-CA3 and posterior spinal cord BCL-2 in two groups of rats (Immunochemistry, magnification, ×40), the yellow arrow is the BCL positive expression site. (**b**) Expression Comparison of rat hippocampus region and posterior spinal cord in two groups (n = 5). **Note:** *P < 0.05.

Based on the analysis of the Bcl-2 staining, the expression of Bcl-2 in the hippocampus and spinal cord of the two groups of rats is shown in Figure (**5**). The anti-apoptotic effect of Bcl-2 was 229 amino acid Bcl-2a, which was located in the inner mitochondrial membrane, the nuclear membrane, and the slippery endoplasmic reticulum. The immunohistochemical staining results revealed the appearance of a few metachromatic particles in the DN group, and the positive expression in the NC group was more yellowish (Figure **5a**). According to Figure (**5b**), the number of anti-apoptotic cells in the hippocampus of the DN group decreased compared with that in the hippocampus of the NC group (NC, n = 4, 0.49 ± 0.03; DN, n = 4, 0.45 ± 0.02; *P* < 0.05), in line with the trends shown in the results for the spinal cord (NC, n = 4, 0.51 ± 0.05; DN, n = 4, 0.44 ± 0.02; *P* < 0.05).

Similarly, from the observation of the Nissl staining under the light microscope, it was seen that in the NC group, the nerve tissue of the posterior funiculus of the spinal cord was complete, tightly arranged, and evenly stained. In the DN group, a large amount of nerve tissue was missing, loosely arranged, or sparse, and the stained area was significantly smaller than that in the NC group, as shown in Figure (**6**).

Finally, we evaluated the changes in rat peripheral nerves under electron microscopy. The myelin sheath of the femoral nerve of the rats in the NC group was observed to be neatly arranged, intact, and dense (Figures **7a** and **b**). In the DN group, the myelin sheath was loosely arranged, broken, or missing and had lost its normal shape; furthermore, a large number of fat cells were grouped around the demyelination (Figures **7c-f**). In the NC group, the unmyelinated fibre capsule was intact, and the nerve microtubules were ordered (Figures **7g** and **h**). In the DN group, the structure of the unmyelinated fibres was disordered, the capsular boundaries were unclear, and the nerve microtubules were disordered (Figures **7i** and **j**). The observation of the femoral nerve *via* electron microscopy revealed demyelination and axonal changes in the peripheral nerve.

### 3.2 Immunohistochemical Staining Results of FIB, GFAP, IBA1, CD31, and Bcl-2

Based on the results of the FIB+ staining, the FIB expression of the two groups of rats revealed the positive expression of FIB to be located mainly in the extracellular matrix; the staining was brown (Figure **8a**). As shown in Figure (**8b**), the positive expression in the NC group was light in colour, and the positive expression in the DN group was significantly higher (NC, n = 5, 0.42 ± 0.06; DN, n = 5, 0.71 ± 0.17; *P* < 0.05).

The presence of GFAP signifies astrocyte activation, which is expressed in the cytoplasm. According to Figure (**8b**), the expression of GFAP in the hippocampus of the DN group increased compared with that in the hippocampus of the NC group (NC, n = 5, 0.53 ± 0.07; DN, n = 5, 0.65 ± 0.04; *P* < 0.05), in line with the trends of the results for the spinal cord (NC, n = 4, 0.58 ± 0.03; DN, n = 4, 0.72 ± 0.07; *P* < 0.05).

Ionised calcium binding adaptor molecule-1 is a microglia-/macrophage-specific protein with increased expression during the activation of these cells. According to Figure (**8b**), the expression of IBA1 in the hippocampus of the DN group increased compared with that in the hippocampus of the NC group (NC, n = 5, 0.62 ± 0.06; DN, n = 5, 0.76 ± 0.06; *P* < 0.05), in line with the trends of the results for the spinal cord (NC, n = 4, 0.69 ± 0.04; DN, n = 4, 0.97 ± 0.05; *P* < 0.05).

Platelet/endothelial cell adhesion molecule-1 is a platelet/endothelial cell adhesion molecule. In this study, its positive expression was located mainly at the junction between the cell membrane and the cell wall between endothelial cells, which allowed the condition of the blood vessels to be observed (Figure **8a**). As shown in Figure (**8b**), there were a few yellow-stained particles in the DN group; the positive expression in the NC group was more yellowish, which was significantly different from that in the DN group (NC, n = 4, 0.49 ± 0.05; DN, n = 4, 0.41 ± 0.03; *P* < 0.05).

### 3.3 Neurodegenerative Changes in T2DM may be caused by the Interaction between FIB and Inflammatory Factors

First, we evaluated the femoral nerve capillaries *via* electron microscopy. In the NC group, the capillary lumen was round, the structure was complete, and the blood vessel wall was smooth (Figures **9a** and **b**). In the DN group, the blood vessels had lost their normal shape; they collapsed, narrowed, twisted, and deformed, and thrombosis was found in the blood vessels (Figures **9c-f**).

Then, we investigated the correlation between DN and FIB in the T2DM rat models. The NCV of the DN group model was used as the dependent variable, and the FIB expression result was used as the independent variable; each variable was assigned the actual measured value. The results showed that FIB expression was closely related to the DN of the T2DM rat models, as shown in Table **1**.

Finally, we investigated the correlation between FIB and the expression of each index. The FIB expression results were taken as dependent variables, and the expression results of GFAP, IBA1, Bcl-2, and CD31 were taken as independent variables; each variable was assigned the actual measured value. The results showed that the expressions of GFAP and IBA1 were closely and positively correlated with FIB, while those of Bcl-2 and CD31 were closely and negatively correlated with FIB, as shown in Table **2**.

## 4 DISCUSSION

The pathological change in DN mainly involves the segmental loss of the myelin sheaths of myelinated nerve fibres, most of which are obvious axonal demyelinations of nerve fibres in the distal limbs [23, 24]. In this study, electrophysiological testing was performed on rats. Compared with the NC group, the femoral nerve motor conduction latency of the T2DM rat models was significantly longer, the amplitude was significantly reduced, and the waveform was discrete, indicating peripheral nerve demyelination and axonal injury. The P40 potential latency of SEP in the lower limbs was significantly prolonged, indicating changes in the central nerve demyelination of the T2DM rat models (Figures **1** and **2**).

**Figure 6:**
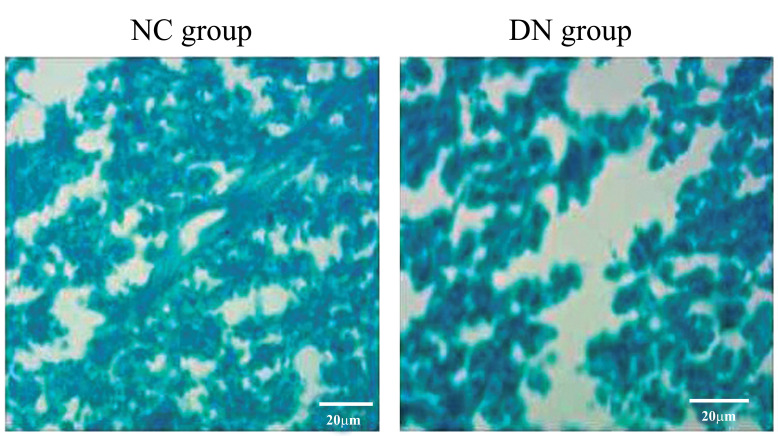
Luxol Fast Blue staining in postspinal cord nerve tissue. magnification, ×100.

**Figure 7:**
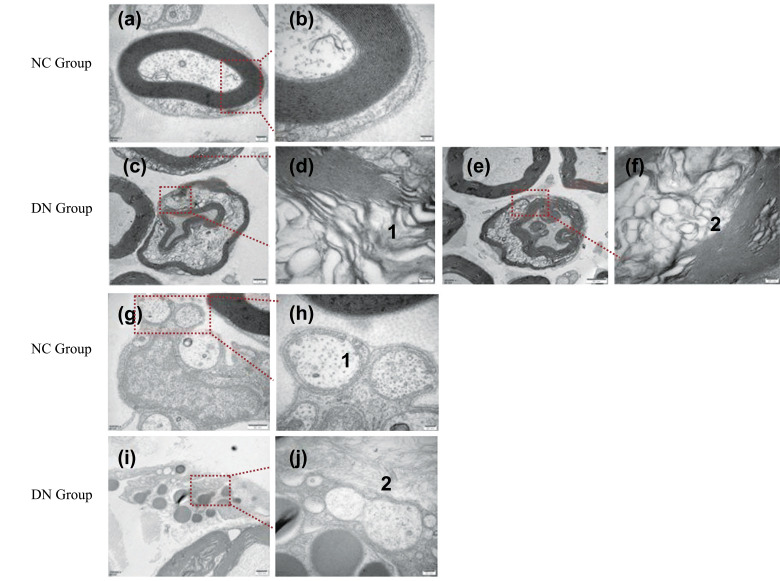
Electron microscopic observation of myelinated and unmyelinated fibers of rat femoral nerve. (**a-b**) The myelinated fiber myelin sheath of the femoral nerve in the NC group. (**c-f**) The myelinated fiber myelin sheath of the femoral nerve in the DN group. **Note:** 1: demyelination; 2: Adipocytes. (**g-h**) The unmyelinated fiber myelin sheath of the femoral nerve in the NC group. **(i-j**) The unmyelinated fiber myelin sheath of the femoral nerve in the DN group. Note: 1: neural microtubules; 2: Nerve capsule. **a, b, f**: (bar = 20nm); **c**: (bar = 1μm); **d**: (bar = 100nm); **e**: (bar = 50nm); **g**: (bar = 50nm); **h, i**: (bar = 20nm); **j**: (bar = 100nm).

**Figure 8:**
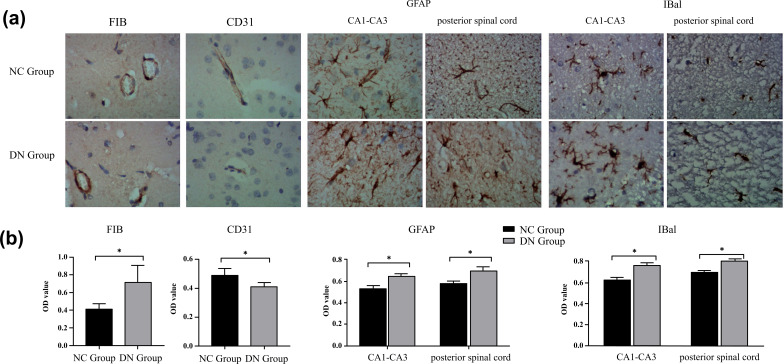
(**a, b**) Immunohistochemical staining of FIB, GFAP, IBa1 and CD31in two groups. magnification, ×40. Scale bar = 20 µm. **Note**: *P < 0.05.

**Figure 9:**
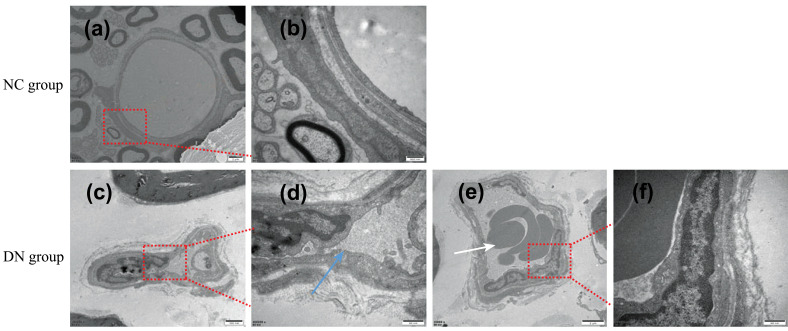
Observation of femoral nerve capillary electron microscope in rats. (**a-b**): femoral nerve capillaries in the NC group. (**c-f**): femoral nerve capillaries in the DN group. White arrow: intravascular blood coagulation; Blue Arrow: collapse of narrow capillaries. **a, e**: (bar = 2μm); **b**: (bar = 500nm); **c**: (bar = 100nm); **d, f**: (bar = 50nm).

The results of the H&E staining, Nissl staining, and Bcl-2 protein expression showed that the neuronal cells in the hippocampus of the T2DM neuropathy rats had undergone harmful changes, including loss, degeneration, and apoptosis (Figures **3-5**). Luxol fast blue staining of the posterior funiculus of the spinal cord showed an obvious loss of nerve fibres (Figure **6**). The observation of femoral nerves under an electron microscope confirmed demyelination and axonal changes in the peripheral nerves, suggesting pathomor-phological changes in the central and peripheral nerves of the rat models (consistent with the electrophysiological nerve changes in the rat models) (Figure **7**). According to the modelling indicators of T2DM neuropathy animal models and the changes in the electrophysiology and histopathology of the rat models, the T2DM neuropathy rat models used in this study were valid.

The results of the immunohistochemistry experiments showed that the positive expressions of FIB, GFAP, and IBA1 in the DN group were stronger than those in the NC group, while the positive expressions of CD31 and Bcl-2 were weaker than those in the NC group (*P* < 0.05), which was consistent with the assumption of this experiment. The statistical correlation analysis suggested that in the T2DM rat models, DN was closely related to FIB deposition. The FIB results had a close positive correlation with the positive expression of inflammatory factors GFAP and IBA1 and a close negative correlation with the positive expression of CD31 and Bcl-2. It is possible that the interaction between FIB and the inflammatory factors regulates the degenerative changes in the nerves in T2DM.

Studies indicate that demyelination occurs in the context of inflammation [25], and the combination of FIB and integrin CD11b/CD18 causes a wide range of cell signal responses, including the mediation of adhesion, migration, chemotaxis, and phagocytosis [26]. Macrophages and activated microglia adhere to partially damaged myelin sheaths and axons and play a destructive role [27]. Fibrinogen stimulates the secretion of macrophage chemokines, such as macrophage inflammatory protein (MIP)-1α, MIP-1β, MIP-2, and monocyte chemoattractant protein-1, while the specific response of macrophages to FIB does not require the conversion of FIB to fibrin [28]. In microglia, FIB mediates the activation of Akt and Rho *via* integrin CD11b/CD18 receptors, leading to cytoskeletal rearrangement and increased phagocytic capacity [29]. Activated microglia can release cytokines, such as IL-1β, IL-6, TNF-α, and chemokines, to promote the apoptosis of nerve cells and oligodendrocytes [30]. Gene knockout or the selection of peptide γ377-395 inhibits the combination of FIB and CD11b, and the clinical symptoms and inflammation of animal models are reduced; however, the selective inhibition of the combination of FIB and integrin CD11b/CD18 does not affect the coagulation function of FIB [29].

Research by Schachtrup *et al.* [31] on astrocytes has shown that in a brain injury model, injecting FIB into the mouse cortex causes astrocytes to proliferate. Fibrinogen can regulate TGF-β-mediated NCS tissue signal transduction and induce reactive astrocytosis and chondroitin sulphate proteoglycan deposition, thereby inhibiting axon growth. The depletion of FIB in mice *via* genetic or drug methods can reduce TGF-β activation, Smad2 phosphorylation, glial cell activation, and nerve (protein) glycan deposition after cortical injury in mice. Therefore, FIB can also be used as an activation signal for astrocytes.

Additionally, FIB can induce ERK1/2 phosphorylation of Schwann cells and upregulation of the p75NGFR apoptosis pathway, control the number of Schwann cells, shut down myelin gene transcription, and inhibit remyelination [32]. In terms of blood vessels, elevated FIB makes the blood hypercoagulable and increases peripheral resistance, causing endothelial cell damage, promoting the infiltration of monocytes/macrophages under the intima, and supporting thrombosis [33]. Furthermore, thrombosis leads to aggregation, reduced blood flow in blood vessels, and blood flow blockage, resulting in ischaemia and hypoxia in the affected area. In this experiment, damage (such as blood vessel thrombosis, collapse, and stenosis of peripheral nerve tissue) could be seen under an electron microscope.

Studies have shown that the co-culturing of FIB and endothelial cells can cause endothelial cells to shrink [34] and increase vascular permeability. Therefore, the increase in FIB in the DN group may have been one of the reasons for the induced endothelial cell damage. In this experiment, the expression of CD31 in the DN group decreased, which is consistent with this study.

The present study has some limitations. Its small sample size may have led to a deviation in experimental results. Additionally, this study was an animal experiment, which requires further research. Notably, the limitation of mobility because of diabetes is a risk factor that increases the risk of amputation as a result of developing ulcers, as shown in multiple clinical and preclinical studies; therefore, we plan to study the potential link between FIB and deformities caused by diabetes [35].

## 5 HIGHLIGHTS

In the type 2 diabetes mellitus (T2DM) neuropathy rats, the expressions of fibrinogen (FIB), glial fibrillary acidic protein (GFAP) and ionised calcium-binding adaptor molecule-1 (IBA1) were upregulated, while the expressions of platelet/endothelial cell adhesion molecule-1 (CD31) and B-cell lymphoma 2 (Bcl-2) were downregulated.

Fibrinogen was positively correlated with the expression of inflammatory factors GFAP and IBA1 and negatively correlated with the expression of CD31 and Bcl-2.

Neurodegenerative changes in T2DM may be caused by the interaction between FIB and inflammatory factors.

## CONCLUSION

Increased levels of FIB may be the cause of neuropathy, the mechanism of which may be related to the promotion of inflammatory response, blood coagulation, and vascular stenosis. Fibrinogen can mediate a wide range of biological effects because its unique structure contains non-overlapping binding motifs of many different receptors; this promotes specific interaction with a large number of integrin and non-integrin receptors expressed on various cells of the haematopoietic, immune and nervous systems. According to the cell distribution of its receptors, it induces different signal transduction pathways, interacts with inflammatory cells, microglia, astrocytes, vascular endothelial cells, Schwann cells, *etc.,* and regulates nerve degeneration and repair. Therefore, selectively regulating the interaction between FIB and its target integrin receptor or using drugs to inhibit or reduce the local deposition of fibrin (*i.e.* FIB) may provide a new clinical treatment direction for neuropathy in patients with T2DM.

## AUTHORS’ CONTRIBUTIONS

(I) Conception and design: Wei- Li Gu and Li Wei

(II) Administrative support: Zhen-Hong Li, Si-Qin Zhang and Pian Ao

(III) Provision of study materials or patients: Xiao-Bei Zhu, Xin Zhao and Xin-Yue Zhang

(IV) Collection and assembly of data: Deng-Feng Zhang, Xiao-Juan Huang

(V) Data analysis and interpretation: Yu Jiang

(VI) Manuscript writing: All authors

(VII) Final approval of manuscript: All authors

## Figures and Tables

**Table 1:** Correlation analysis of NCV and FIB in rat model.

**Index**	**Relevance**	***P***
FIB	0.928**	0.001

**Note:** **P < 0.01.

**Table 2:** Analysis of the correlation between expression and FIB of indicators.

**Index**	**Relevance**	***P***
GFAP	0.731*	0.040
IBA1	0.786*	0.021
BCL-2	-0.743*	0.035
CD31	-0.795*	0.018

**Note:** *P < 0.05.

## Data Availability

All data generated or analysed during this study are included in this article. Further enquiries can be directed to the corresponding author.

## LIST OF ABBREVIATIONS

Bcl-2: B-cell Lymphoma-2
CD31: Platelet/Endothelial Cell Adhesion Molecule 1
CMAP: Compound Muscle Potential Amplitude
CSPG: Chondroitin Sulphate Proteoglycans
DML: Distal Movement Latency
DN group: T2DM Neuropathy Model Group
DN: Diabetic Neuropathy
FIB: Fibrinogen
GFAP: Glial Fibrillary Acidic Protein
HE: Hematoxylin and Eosin
IBA1: Ionized Calcium-binding Adaptor Molecule 1
LTP: Long-term Potentiation
MCV: Motor Conduction Velocity
MNAP: Motor Nerve Action Potential
NC group: Control Group
NCV: Nerve Conduction Velocities
SD: Sprague-Dawley
SEP: Somatosensory-evoked Potential
STZ: Streptozotocin
T2DM: Type 2 Diabetes Mellitus
